# Comparing Adsorption of an Electron-Rich Triphenylene Derivative: Metallic vs Graphitic Surfaces

**DOI:** 10.1021/acs.jpcc.4c02376

**Published:** 2024-06-19

**Authors:** Joris de la Rie, Qiankun Wang, Mihaela Enache, Milan Kivala, Meike Stöhr

**Affiliations:** † Zernike Institute for Advanced Materials, University of Groningen, Nijenborgh 4, 9747 Groningen, AG, The Netherlands; ‡ Institute of Organic Chemistry, Heidelberg University, Im Neuenheimer Feld 270, 69120 Heidelberg, Germany

## Abstract

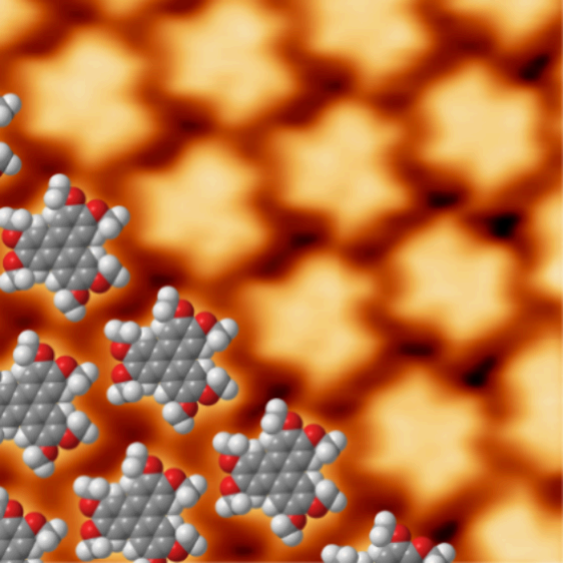

Crucial to the performance of devices based on organic molecules is an understanding of how the substrate–molecule interface influences both structural and electronic properties of the molecular layers. Within this context we studied the self-assembly of an alkoxy-triphenylene derived electron donor (HAT) in the monolayer regime on graphene/Ni(111). The molecules assembled into a close-packed hexagonal network commensurate with the graphene layer. Despite the commensurate structure, the HAT molecules only had a weak, physisorptive interaction with the substrate as pointed out by the photoelectron spectroscopy data. We discuss these findings in view of our recent reports for HAT adsorbed on Ag(111) and graphene/Ir(111). For all three substrates HAT adopts a similar close-packed hexagonal structure commensurate with the substrate while being physisorbed. The ionization potential is equal for all three substrates, supporting weak molecule–substrate interactions. These findings are remarkable, as commensurate overlayers usually only form at strongly interacting interfaces. We discuss potential reasons for this particular behavior of HAT which clearly sets itself apart from most studied molecule–substrate systems. In particular, these are the relatively weak but flexible intermolecular interactions, the molecular symmetry matching that of the substrate, and the comparatively weak but directional molecule–substrate interactions.

## Introduction

Organic semiconductors are lightweight, are usually stable in air, and can be processed at low cost, and molecular properties can be efficiently tuned by chemical design of the molecules used. This high degree of versatility makes devices based on organic molecules show great promise for the next generation of electronic devices, especially in the fields of photovoltaics^1,2^ and opto-electronics^3,4^—applications that benefit greatly from the tunability of energy levels in organic molecules. Crucial to the functionality of organic semiconductors is the organic–inorganic substrate interface, the structural and electronic properties of which often influence the entire film.^2,3,5−10^ Strong molecule–substrate interactions can induce a molecular ordering for the first molecular layer that is far from the 3D molecular crystal structure which makes the desired layer by layer growth difficult,^11−13^ but even if the monolayer order is close to that of a certain face of the bulk crystal layer by layer growth is not guaranteed.^12,14−16^ When it comes to the electronic properties of molecular films, it is advised to study the film growth beginning with the first layer and then continuing gradually to disentangle the contributions of interface effects (e.g., band bending, charge transfer, induced interface states, screening, and interface dipoles) from bulk properties.^5,8,17,18^ Therefore, the study of structural and electronic properties of thin molecular films, starting from the submonolayer regime, remains a topic of considerable interest to date. Recently, particular attention has been given to strategies reducing the interactions at the metal–organic interface to facilitate layer by layer growth, for example by insertion of buffer layers and tailoring of the molecules, respectively.^13,18−22^

One of the strategies developed to reduce the interactions between the molecular film and its support is the insertion of an inert 2D material. Graphene, perhaps the most studied 2D material, has been successfully shown to prevent decomposition of organic molecules on reactive surfaces^23,24^ and inhibit charge transfer.^25^ Even if the decoupling is not fully achieved—it is most effective when the graphene–substrate interactions are also weak^26,27^—a graphene buffer layer does efficiently weaken the interface interactions.^27−32^ Similar effects have been reported for other 2D materials, such as hexagonal boron nitride,^20^ MoS_2_,^33^ and others.^34−36^ Such buffer layers can also modify the molecular orientation compared to the molecular orientation without a buffer layer. This change in orientation can be limited to the first adsorbed layer but also extended to further layers,^32,37,38^ which in turn further influences the electronic properties of the molecular film.^19,37^

High-quality graphene can be grown on various transition metal surfaces by means of chemical vapor deposition, using primarily ethylene (C_2_H_4_). As the precursor decomposition is typically limited once the catalytic metal surface is covered by a complete graphene layer, growth is easily limited to a monolayer.^39,40^ For most transition metals, increasing corrugation and variation of electronic properties across the Moiré pattern goes hand in hand with increasing metal–graphene interaction.^41^ The case of graphene grown on Ni(111) (Gr/Ni(111)) is an outlier when it comes to this trend as the graphene–metal interaction is strong but no Moiré pattern is formed. The small lattice mismatch results in a 1 × 1 commensurate structure and graphene being adsorbed only 2.1 Å above the nickel surface.^42^ Simultaneously, this structure leads to a strong hybridization between the graphene π* band and Ni 3d orbitals, shifting the Dirac point more than 2 eV below the Fermi level and opening a band gap at the K point.^43^ This places Gr/Ni(111) in an odd position compared to graphene on other transition metals: structurally close to free-standing graphene but electronically strongly distorted.

**Scheme 1:**
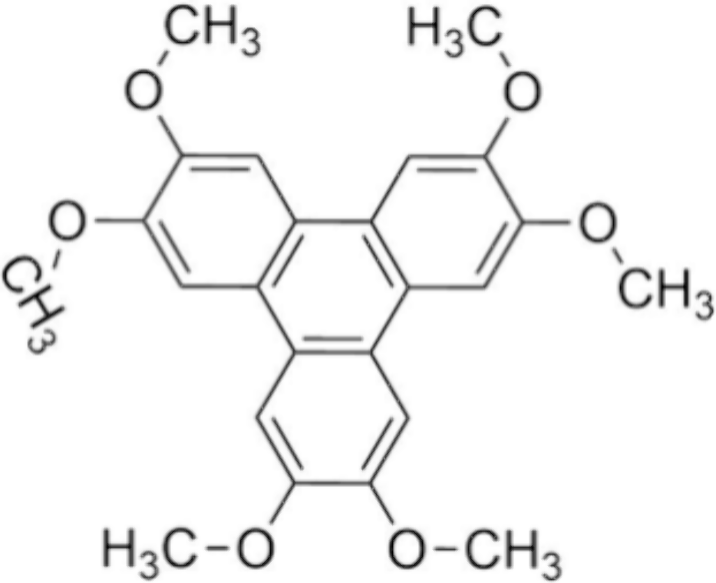
Molecular Structure of 2,3,6,7,10,11-Hexamethoxytriphenylene (HAT)

Triphenylene derivatives are a versatile set of molecules, as the polycyclic aromatic triphenylene backbone is readily functionalized with varying moieties^44−46^ which can transform it into both an electron acceptor^47^ and donor.^48^ Despite this versatility, most studies of surface-supported self-assembled triphenylene derivatives focus on either the strong acceptor 2,3,6,7,10,11-hexacyanohexaazatriphenylene (HATCN)^49−52^ or the unsubstituted triphenylene.^53,54^ Here, we focus on the electron donor^55,56^ molecule 2,3,6,7,10,11-hexamethoxytriphenylene (HAT), which consists of a planar triphenylene backbone functionalized with six methoxy groups (Scheme 1). HAT has recently been studied in the monolayer regime, on Ag(111)^21^ and graphene on Ir(111) (Gr/Ir(111)).^22^

Herein we report on the self-assembly of HAT on Gr/Ni(111), studied by scanning tunneling microscopy (STM) and low-energy electron diffraction (LEED) to obtain knowledge of the molecular ordering and by X-ray and ultraviolet photoelectron spectroscopy (XPS and UPS) to gain insight in the electronic properties of the HAT/Gr/Ni(111) interface. We also compare our findings to previous studies of HAT on Ag(111)^21^ and Gr/Ir(111).^22^

## Methods

### Synthesis

HAT was synthesized upon oxidative cyclotrimerization of 1,2-dimethoxybenzene with iron(III) chloride in nitromethane and CH_2_Cl_2_ at 0 °C for 30 min by following the procedures reported for related alkoxy-substituted triphenylene derivatives previously.^44,46^ After quenching the reaction with methanol and washing the formed precipitate with methanol and water, HAT was obtained in 37% yield as a gray amorphous solid.

### Sample Preparation

The Gr/Ni(111) substrate was prepared in a two-chamber ultrahigh vacuum (UHV) system (a preparation chamber having a base pressure below 10^–9^ mbar and a measurement chamber with base pressure lower than 5 × 10^–10^ mbar). The Ni(111) crystal was cleaned by cycles of argon ion sputtering and annealing at 500 °C. Chemical vapor deposition was used to prepare single-layer epitaxial graphene by exposing the Ni(111) single crystal to a partial ethylene pressure of 5 × 10^–6^ mbar for 10 min while being held at 500 °C. HAT molecules were sublimed from a Knudsen cell evaporator (CreaPhys), while the Gr/Ni(111) substrate was held at room temperature. A water-cooled quartz crystal microbalance was used to monitor the deposition rate.

### Measurements

Measurements were carried out in the analysis chamber of the aforementioned UHV system at room temperature. The analysis chamber houses a hemispherical analyzer (Prevac EA15), twin anode X-ray source (Prevac RS 40B1), He discharge lamp (Prevac UVS 40A2), variable-temperature STM (Scienta Omicron GmbH), and LEED optics (SPECS). Photoemission spectra were acquired using Al Kα (1486.7 eV) (XPS) and He I (21.2 eV) and He II (40.8 eV) radiation at normal emission (UPS). For secondary electron cutoff (SECO) measurements, the sample was biased to −5 V. STM images were recorded with Pt/Ir tips (mechanically pulled) in constant current mode. Bias voltages are reported with respect to the sample, which is grounded. Images were processed using WSxM.^57^ LEEDpat was used to simulate LEED patterns.^58^

## Results

**Figure 1:**
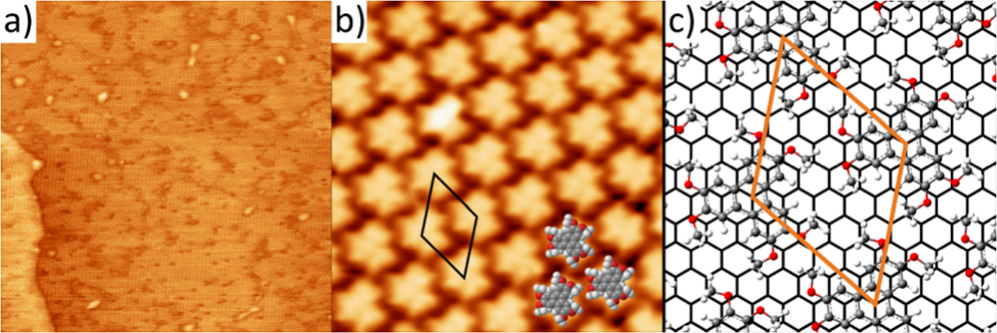
Structure of HAT on Gr/Ni(111). (a) Overview STM image of 0.9 monolayer HAT on Gr/Ni(111) (100 × 100 nm^2^, 1.56 V, 20 pA). (b) High-resolution STM image of 0.9 monolayer of HAT on Gr/Ni(111) (10 × 10 nm^2^, −1.8 V, 41 pA). A black diamond indicates the unit cell. The molecular model of HAT is overlaid to guide the eye. (c) Tentative model of HAT on graphene on Ni(111). The graphene lattice is indicated by the black hexagons and the unit cell of HAT by the orange diamond.

In Figure 1, STM data for the self-assembly of HAT on Gr/Ni(111) are presented. Figure 1a shows a typical large-scale STM image for close to monolayer coverage (0.9 monolayer) of HAT deposited on Gr/Ni(111). As can be seen from the STM image, the surface is covered by small islands of HAT molecules with typical diameters of tens of nanometers. The islands are broken up by graphene growth defects (identified as bright white dots in the STM images, see also Figure S2 in the Supporting Information) and the step edges of the Ni(111) substrate. Missing molecule defects were found both at the island edges and in the center of the islands. Within the islands, the HAT molecules are arranged in a close-packed hexagonal network. The islands form in two mirror domains rotated by ± 19° with respect to the graphene lattice (more information is presented in Figure S1 in the Supporting Information). A close-up image of one of the islands is shown in Figure 1b. The shape of the individual molecules resembles a six-pointed star. The center of the star we identify with the triphenylene backbone and the points with the methoxy groups, respectively. From our STM and LEED data (see Figures S3, S4, and S5 in the Supporting Information) we measure the rhombic unit cell to have size *a* = *b* = (1.30 ± 0.04) nm with an enclosing angle of θ = (120 ± 0.5)°. The self-assembled network is rotated ±(19 ± 0.5) ° with respect to the Gr/Ni(111) substrate. This corresponds to a commensurate superstructure with an epitaxial matrix of , which can also be expressed in Wood’s notation as a 2√7 × 2√7 R19.1° overlayer. The tentative structure model is displayed in Figure 1c. The close-packed hexagonal network is stabilized by hydrogen bonds between the methoxy groups, with the oxygen atoms of one molecule forming hydrogen bonds with the hydrogen atoms of the methoxy groups of the neighboring molecules.

**Figure 2:**
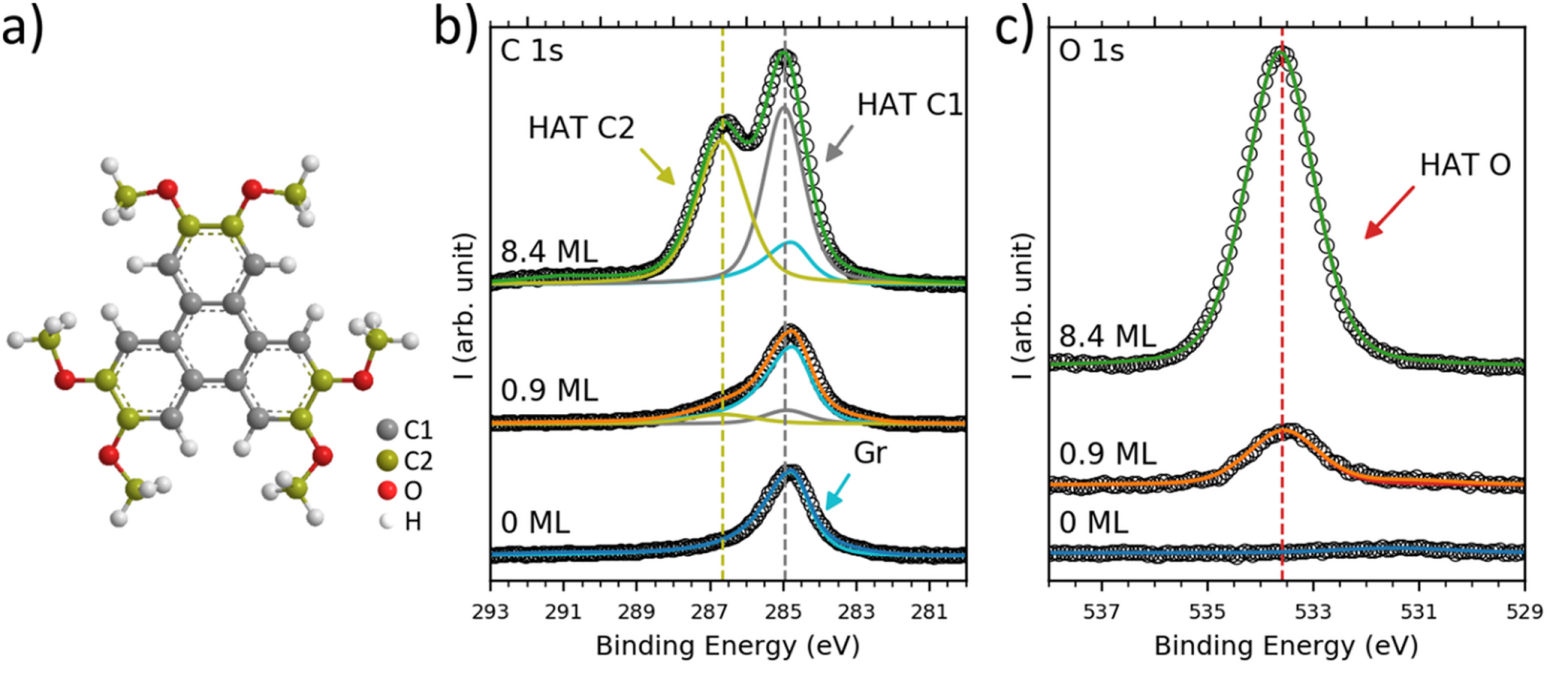
(a) Ball-and-stick model of HAT, with the two carbon species distinguished in the XPS analysis in gray (HAT C1) and yellow (HAT C2). (b) XPS spectra of the C1s core level for Gr/Ni(111) (blue), monolayer HAT (orange), and multilayer HAT (green). For clarity, only the three main peaks used in the fit are shown here. These are the graphene peak (cyan, all three spectra) and the HAT C1 and C2 peaks (gray and yellow, 0.9 and 8.4 ML spectra). (c) O 1s core level spectra for Gr/Ni(111) (blue), monolayer HAT (orange), and multilayer HAT (green). Only the main peak (oxygen in HAT) is shown here (red, position indicated by the dashed red line). For both the C 1s and O 1s spectra, more information on the fit is presented in the Supporting Information (Figures S6 and S7, Table S1).

In order to study the interface interactions (such as charge transfer or chemical bonding) between HAT and graphene, we performed C 1s and O 1s core level XPS measurements, both shown in Figure 2. We first discuss the carbon 1s core level data, shown in Figure 2b. The C 1s spectrum for Gr/Ni(111) consists of a single peak, and as a monolayer of HAT is deposited a shoulder appears at higher binding energy; for the multilayer spectrum two peaks are clearly resolved. The C 1s spectra were all fitted with a graphene peak at 284.8 eV (to obtain a good fit, it was necessary to include a small carbide peak at 283.4 eV as well as a π–π* shakeup satellite at 291.0 eV,^59,60^ see Figure S6 in the Supporting Information). For the HAT molecule, we fitted two peaks with equal area: one for the carbon atoms bound to only hydrogen and carbon (284.9 eV, labeled C1, gray atoms in Figure 2a) and one for the carbon atoms bound to oxygen (286.7 eV, labeled C2, yellow in Figure 2a). This might seem counterintuitive as there are four chemically distinct carbon environments in HAT. However, given the similarities for certain environments, which is equal to tiny experimentally not resolvable chemical shifts, we grouped these four chemically different C atoms into two groups. In particular, the C atoms bound to three other C atoms and the C atoms bound to two C atoms and one H atom contribute to the C1 peak, while the C2 peak has contributions from the C atoms bound to one O atom and either 2 C atoms or 3 H atoms. Since all six oxygen atoms in HAT are in the same chemical environment (marked red in Figure 2a), the O 1s spectrum is fitted well by a single molecular peak (at 533.6 eV). We would like to point out that we cannot exclude a minor contribution to the O 1s peak from the sample holder at lower binding energy (see Figure S7). The details of the fits can be found in the Supporting Information in Table S1. Taken together, the C 1s and O 1s spectra point toward HAT being physisorbed on Gr/Ni(111). The only difference between the monolayer and multilayer spectra is a shift of the molecular peaks to higher binding energy by less than 0.1 eV (see Table S1) for the multilayer. This shift could be assigned to a reduction in core–hole screening, but the shifts are too small to be unambiguously ascertained. In fact, the most notable feature of the XP spectra is the almost complete independence of the spectra (both peak position and peak width) of film thickness. The absence of any interface peaks excludes the formation of a chemical bond between the molecules and substrate. Hence, we deduce that HAT is physisorbed on the Gr/Ni(111) substrate.

**Figure 3:**
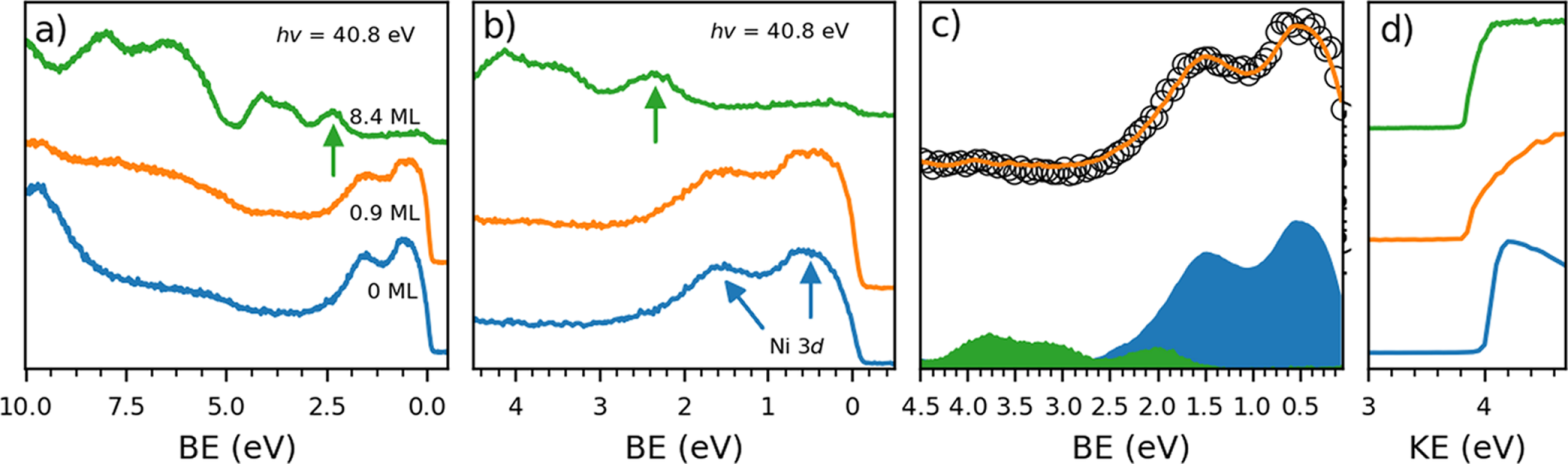
UPS data for HAT on Gr/Ni(111): pristine graphene on Ni(111) (blue), 0.9 ML HAT (orange), and 8.4 ML HAT (green). (a) Valence band spectra and (b) close up of the HOMO region. The green arrow points out the HAT HOMO for the multilayer spectrum (at 2.3 eV) and the blue arrows the Ni 3d bands. Overlap between the Ni 3d bands and the HAT HOMO prevents easy identification of the HAT HOMO for the monolayer spectrum, so we fit the monolayer spectrum with a combination of the Gr/Ni(111) and multilayer HAT spectra. (c) Fit of the monolayer spectrum. The measured monolayer spectrum is shown by the open circles, the fit by the orange curve. Filled blue and green curves give the contributions to the fit from the graphene (85%) and multilayer (15%, 0.35 eV shifted to lower binding energy) spectra, respectively. This allows us to identify the HAT HOMO at 2.0 eV for the monolayer spectrum. (d) Secondary electron cutoff.

To gain more insight into the energy level alignment at the interface, we carried out UPS measurements. In Figure 3 we compare spectra of the Gr/Ni(111) substrate, a monolayer, and a multilayer of HAT. Identification of the highest occupied molecular orbital (HOMO) of HAT is straightforward for the multilayer spectrum, where it is located at 2.3 eV (marked by a green arrow). For the monolayer spectrum, the identification of the HOMO level is more difficult, as the HOMO position most likely overlaps with the Ni 3d bands, which would preclude the identification of the HOMO position independent of the attainable resolution (Figure 3a). To nevertheless obtain the energy position of the HAT HOMO, we fitted the monolayer spectrum with a combination of the substrate spectrum and the energy-shifted multilayer spectrum. A good fit reproducing all features of the monolayer spectrum is produced for a combination of 85% Gr/Ni(111) spectrum and 15% multilayer HAT spectrum shifted by 0.35 eV toward lower binding energy (see Figure 3b and 3c). This places the HAT HOMO at 2.0 eV for the monolayer case. As also the HOMO-1 and HOMO-2 levels are included in the fitted energy range, it becomes obvious that all (fitted) HOMO levels shift rigidly to lower binding energy compared to the multilayer case, which can be attributed to reduced core–hole screening for molecular layers farther away from the metal substrate. However, as such a shift could also be caused by the presence of molecular dipoles, we investigated the work function of the molecular films for more information.

The work function (Figure 3d) was obtained by measuring the secondary electron cutoff (SECO) at low kinetic energy in the UPS spectra. For Gr/Ni(111) we measured a work function of 4.0 eV (a typical value when compared to the literature^25,29^), which decreased to 3.8 eV after deposition of a monolayer of HAT. Deposition of additional layers of HAT did not result in a further change of the work function. An interface dipole formed by electron transfer from the first layer of HAT into the substrate cannot explain these results as this would give rise to distinct interface peaks in the XP and UP spectra. An intrinsic dipole of the HAT molecule can also be excluded because this would result in a further decrease in the work function when the second and further layers are deposited and moreover is at odds with the planar adsorption geometry we observed for the first layer (see below). We thus conclude that the work function is reduced by the push back of the substrate electron cloud (also known as Pauli repulsion or pillow effect) by the first layer of molecules, while the shift of the HOMO levels is due to core–hole screening. Taken together, the XPS and UPS measurements point to a weak, physisorptive interaction between the HAT molecules and the Gr/Ni(111) substrate.

If HAT is indeed physisorbed on each substrate and no charge transfer occurs, the ionization potential (IP, the minimum energy necessary to extract one electron from a molecule) of HAT should be equal on all three substrates. If charge transfer occurs, the amount of charge transfer would vary depending on the substrate work function. If that were the case, the value of the HAT IP should depend on the substrate (known also as Fermi level pinning). However, if the IP is the same on each substrate, it is likely that no integer charge transfer occurs.^5,8,61^ The IP is the sum of the monolayer work function Φ_mono_ and the molecular HOMO binding energy. Note that we use the work function after deposition of a HAT monolayer rather than the work function for the pristine substrate (Gr/Ni(111)) as would be usually done. This is necessary because our samples are prepared and measured *in situ*, and thus, the pillow effect (or push-back effect) only sets in upon deposition of organic molecules on Gr/Ni(111). However, the pillow effect must be accounted for because of its substrate dependence. Thus, the work function of the monolayer HAT samples needs to be used as a reference to verify whether the HAT IP is constant across substrates. In the case that the pillow effect is the only contribution to the change in work function of the molecule-covered substrate (as compared to the pristine substrate), the ionization potential of HAT will be the same on each substrate.^5,62^

The parameters relevant for verifying a constant ionization potential are presented in Table 1. As expected, the work function shift upon adsorption of HAT is substrate dependent: ΔΦ = 0.2 eV for both Gr/Ni(111) and Gr/Ir(111) and ΔΦ = 0.8 eV for Ag(111). The work function for a ML of HAT varies from 3.7 eV (Ag(111)) to 4.4 eV (Gr/Ir(111)) with that for Gr/Ni(111) in between at 3.8 eV. The binding energy of the HAT HOMO is similarly spread, ranging from a maximum of 2.2 eV (Ag(111)) to 1.5 eV (Gr/Ir(111)), again with the value on Gr/Ni(111) between these being 2.0 eV. Adding up the work function and HOMO position gives the HAT IP which ranges from 5.8 to 5.9 eV across the three substrates—which agrees well within our experimental resolution of ±0.1 eV for both the work function and HOMO position. A comparison across the three substrates thus reveals a constant IP for HAT as it fits with an absence of charge transfer between HAT and each substrate. Again, together with the UPS and XPS data, this points to the molecules being physisorbed on the three substrates Gr/Ni(111), Gr/Ir(111), and Ag(111) with no integer charge transfer occurring.

**Table 1:** Parameters Relevant to the HAT Ionization Potential on Each Substrate: Substrate Work Function, Work Function after Deposition of 1 ML of HAT, Position of the HAT HOMO for 1 ML, and HAT Ionization Potential for 1 ML^a^

	Substrate work function (eV)	ML HAT HOMO (eV)	ML HAT work function (eV)	ML HAT IP (eV)
Gr/Ni(111)	4.0	2.0	3.8	5.8
Gr/Ir(111)	4.6	1.5	4.4	5.9
Ag(111)	4.5	2.2	3.7	5.9

a Data for Gr/Ni(111) are taken from this publication and for Gr/Ir(111) from ref (22). The position of the HOMO for Ag(111) is taken from ref (21), and the work function data are shown in the Supporting Information, Figure S9.

## Discussion

In this section, we compare our current results for HAT on Gr/Ni(111) to recent publications on HAT/Ag(111)^21^ and HAT/Gr/Ir(111).^22^ The aim is to shine light on the apparent contradiction that HAT interacts only weakly with each substrate, while at the same time it forms a commensurate structure on all three substrates.

On each of the three substrates, HAT formed a close-packed hexagonal network. Within this network the molecules arrange in the same manner, namely by hydrogen bonds. The lattice parameter varies slightly (1.30 nm on Gr/Ni(111) and Gr/Ir(111), 1.32 nm on Ag(111)) as does the rotation angle of the HAT network with respect to the substrate (19° on Gr/Ni(111) and Gr/Ir(111), 11° on Ag(111)). These minor differences serve to make the HAT network commensurate with the different substrate lattices (graphene has a honeycomb unit cell with a lattice parameter of 0.25 nm and Ag(111) a hexagonal unit cell with lattice parameter 0.29 nm). The only difference we observed with respect to structure was the size of the molecular islands. On Ag(111) and Gr/Ir(111), HAT formed islands that extend for hundreds of nanometers with few defects, mostly (single) molecular vacancies. This is in contrast to the assembly on Gr/Ni(111), where the islands were only tens of nanometers large and molecule vacancies were more common. This is likely due to the comparatively high defect density of graphene on the Ni(111) substrate (*vide supra*, compared to a much lower defect density on Gr/Ir(111)^22^). These defects hinder the diffusion of the HAT molecules and thereby give rise to smaller islands and a higher density of molecular vacancies.

Next, we discuss the electronic properties of HAT as derived from XPS and UPS measurements. The XPS spectra for each substrate were fitted with two peaks for molecular carbon and one for molecular oxygen, so results are comparable across substrates. For the three different substrates, the reported values for each peak are highly similar, for both monolayer and multilayer samples. The location of the HOMO position as derived from the UPS measurements differs between the three substrates, but that is to be expected for a weakly adsorbed molecule on substrates with differing work functions. In fact, the ionization potential of HAT is the same on each substrate, which underpins the similarities for HAT adsorbed on each substrate. Taken all together, the experimental data for HAT on Ag(111), Gr/Ir(111), and Ni(111) point to HAT being physisorbed on all three substrates, with no chemical bonding or charge transfer occurring.

Combining the information given above and the respective conclusions drawn, we conclude that HAT is clearly physisorbed on the three different substrates investigated. That is generally interpreted in the way that HAT favors intermolecular interactions over molecule–substrate ones. We identified that HAT forms a commensurate overlayer on Ag(111), Gr/Ir(111), and Gr/Ni(111) which is typically a sign that molecule–substrate interactions are not negligible (often a chemisorption is present) and thus are relevant for the structure formation. These two findings are at first glance in contrast to each other as well as to the behavior usually observed at metal–organic interfaces. Weakly interacting (physisorbed) molecules tend to form incommensurate structures,^63−66^ and commensurate structures are normally formed at strongly interacting (chemisorbed) interfaces.^67−69^ At first glance, it might therefore seem that the HAT overlayers are actually not commensurate but only appear to be so due to limited experimental resolution. This is unlikely as the commensurability on each substrate is derived from both STM and LEED measurements (and in the case of Ag(111) supported by DFT calculations as well). Additionally, if there were no significant substrate–molecule interactions we would likely observe different rotational domains instead of a single domain and its equivalent mirror domain (mirrored at a principal substrate direction).

We propose that this unusual behavior mainly arises from the unique properties of the HAT molecule: its symmetry and large aromatic backbone make it well suited to optimize π–π interactions with graphene, while the hydrogen bonds between methoxy groups that stabilize the self-assembled network are weaker in comparison to the hydrogen bonds formed between other commonly studied molecules.^70^ Importantly, since the H-bonds between individual HAT molecules exhibit a certain amount of directional flexibility due to the rotational adaptability of the methoxy group,^21^ a greater degree of rotational flexibility of the HAT molecules within the hexagonal network is achieved, enabling the adaptation of the unit cell parameters to the underlying substrate for obtaining a commensurate arrangement. Accordingly, the HAT network can optimize its interaction with the substrate through π–π interactions without weakening the intermolecular bonds. While these conditions might seem rather easily met, we show below why most molecules so far studied on graphene fail to meet them and how this hinders the formation of commensurate self-assembled monolayers.

At first, it is necessary to look at the molecule–substrate interactions that drive the formation of a commensurate structure. π–π interactions are attractive interactions between aromatic molecules that promote a parallel adsorption geometry, but these interactions are unlikely to drive a commensurate adsorption.^71,72^ A directional interaction could be found between the oxygen moieties of the molecules and the π-system of graphene. These interactions are site-specific^73^ and thus could drive the commensurate adsorption of HAT. When designing a molecule to take advantage of the π–π interactions, it is therefore important to take into account the functional groups attached to the molecule and their place on the aromatic backbone—which together dictate the symmetry of the molecule and the self-assembled network it might form on graphene.

The importance of these factors can be seen for the frequently studied perylene derivatives PTCDA (perylenetetracarboxylic dianhydride) and perylenetetracarboxylic diimide (perylenetetracarboxylic diimide), which individually adsorb in a geometry that aligns the perylene backbone with graphene. However, the 2-fold symmetric herringbone network these molecules form turns the molecules away from this orientation, increasing intermolecular hydrogen bonds between their functional groups at the cost of reducing the π–π interaction.^74,75^ Similar arguments are likely at play for other commonly studied molecules such as 7,7,8,8-tetracyanoquinodimethane^76,77^ derivatives,^78^ functionalized porphyrins,^79^ and phthalocyanines:^80^ the 2- and 4-fold symmetry of these molecules together with their desire to form intermolecular interactions between their functional groups lead to a competition of intermolecular and π–π interactions, often hindering the formation of a commensurate superstructure.

Another set of widely studied molecules can be found in carboxylic acid-functionalized benzene derivatives, such as trimesic acid (TMA) or 1,3,5-benzenetribenzoic acid (BTB). These molecules consist of a central benzene ring, functionalized in a 3-fold symmetric manner with three carboxylic acid groups (TMA) or carboxyphenyl groups (BTB). Both molecules have the right symmetry as well as an aromatic backbone and therefore should be able to form a commensurate overlayer on graphene, optimizing both intermolecular and π–π interactions. This is not the case, however, as BTB forms incommensurate structures on Gr/Cu(111)^24^ and Gr/Ir(111).^81^ Initial reports claimed TMA forms a commensurate network on graphene on Cu(111),^82^ but later publications showed that—while close to commensurate—the network is actually incommensurate.^83^

The reason for this can be found in the strong, dimeric hydrogen bonds formed between the carboxylic acid (COOH) groups, which are among the strongest and most directional hydrogen bonds commonly found in self-assembled monolayers.^84−87^ Both the strength and directionality of these hydrogen bonds means that intermolecular interactions dominate over molecule–substrate interactions.^82,85^ Because of this, TMA and BTB networks lack the flexibility to form a commensurate structure. The high strength and directionality of carboxylic acid dimers can also be seen in direct comparison to other molecules, e.g., by comparing self-assembly of TMA and benzene-1,3,5-tricarboxaldehyde (TCA). While structurally very similar, the aldehyde and carboxylic acid group give rise to very different intermolecular bonds.^70^ This can be seen in the self-assembled network formed by TCA: its close-packed hexagonal network is more similar to the network formed by HAT than TMA,^86^ and the intermolecular interactions are far weaker compared to TMA.^88^ Given its structure, TCA would be an ideal molecule to test these predictions, but unfortunately no studies to date have investigated whether TCA forms commensurate overlayers on graphene.

The above arguments can nicely explain the commensurability of HAT on Gr/Ni(111) and Gr/Ir(111) but cannot completely explain the case of HAT/Ag(111). The arguments regarding intermolecular bond strength and flexibility apply equally to HAT/Ag(111) and HAT/Gr, but on the metal surface, π–π interactions cannot be the driving force behind the commensurate structure. Instead, however, π–metal interactions result in an attractive force and have been reported to drive a preferential adsorption site for the molecules.^89−91^ Furthermore, it has been shown that oxygen-containing moieties lead to charge rearrangement in the molecule that leaves its aromatic backbone polarized with respect to the oxygen-containing moieties. This allows it to interact with the metal surface via dispersion forces, independent of bonds potentially formed by the oxygen atoms.^16,92^ As HAT has six oxygen-containing groups arranged in a 3-fold symmetric manner—the six methoxy groups, which tend to donate electrons to polycyclic aromatic molecules^93−95^—this would likely result in a positive, directional interaction at the HAT/Ag(111) interface similar to the π–π interactions at the HAT/graphene interfaces.

**Figure 4:**
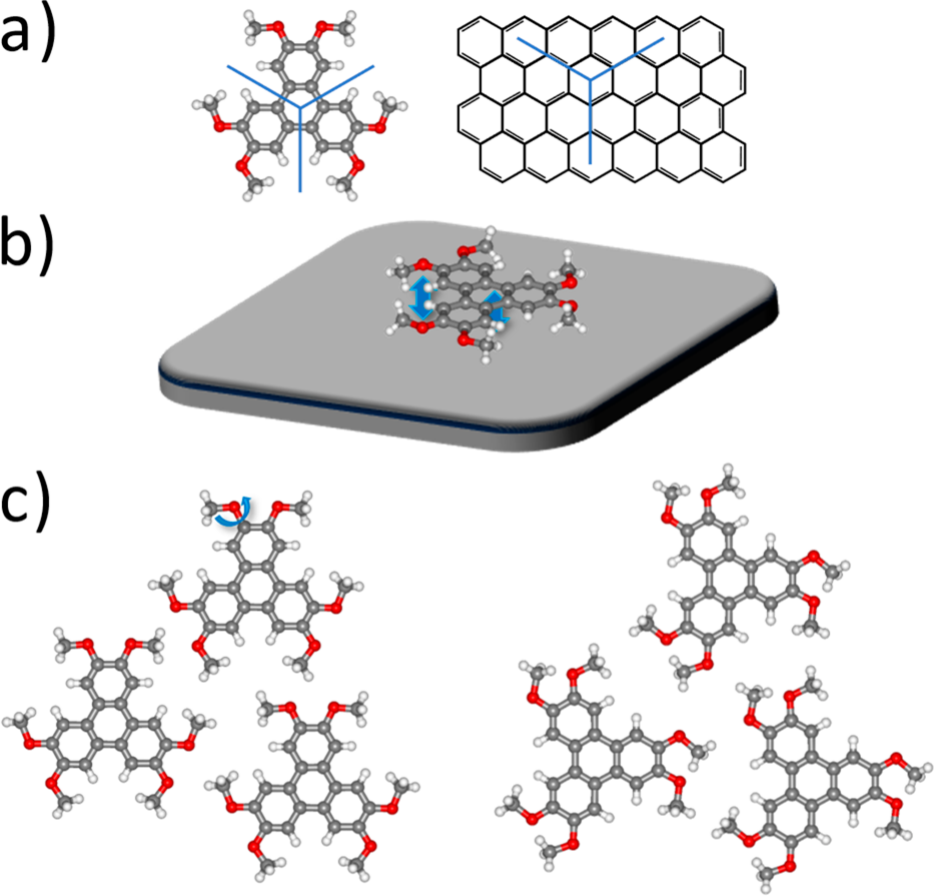
Properties of the HAT/graphene and HAT/Ag(111) interfaces that drive epitaxial growth despite weak molecule–substrate interactions. (a) Matching symmetry of the molecule and substrate. (b) Site-specific, directional molecule–substrate interactions. (c) Rotational adaptability of the HAT methoxy groups.

The main findings of our discussion are summarized in Figure 4.

## Summary and Conclusions

In the present study, we report on the self-assembly of the triphenylene-based electron donor HAT on graphene/Ni(111). From STM and LEED data it was derived that the HAT molecules assembled in a close-packed hexagonal structure that is commensurate with the graphene substrate. Investigation of the electronic structure by XPS and UPS showed that the HAT molecules were physisorbed on the surface.

The finding of a commensurate structure at a weakly interacting interface was surprising since it goes against the established trend for molecules self-assembled on metal surfaces requiring a substantial molecule–substrate interaction for commensurability. However, similar findings were reported for HAT on Ag(111)^21^ and graphene/Ir(111).^22^ Comparison of the HAT ionization potential reveals that it is constant across all three substrates, which points to a weakly interacting interface in each case.

We rationalize these findings from the (chemical) structure of the HAT molecule. The hydrogen bonds that stabilize the close-packed hexagonal network are relatively weak while offering flexible directionality compared to those usually occurring in self-assembled monolayers. Simultaneously, the triphenylene backbone gives the molecule an extended π system which helps drive the formation of a commensurate structure through π–π interactions with graphene. On Ag(111), the molecule–substrate interaction is most likely based on the positive polarization of the triphenylene core by the methoxy groups. The combination of flexible intermolecular interactions, weak physisorptive molecule–substrate interactions, and a molecular symmetry which matches that of the substrate is what leads in the present case to the formation of a commensurate overlayer. These findings present new options in designing molecules for organic electronics in view of tuning growth mode as well as preserving possible molecular functionalities. In particular, triphenylene derivatives are a promising class of molecules for further research in this direction, given that their large aromatic backbone and 3-fold symmetry match well with graphitic interfaces. Additionally, the great versatility in functional groups that are readily attached to the triphenylene core enables the modifícation of electron donating/withdrawing properties and intermolecular interactions.

## Supporting Information Available

Additional STM, LEED, and UPS (He I light) data for HAT/gr/Ni(111), XPS C 1s and O 1s data including all fitted peaks and parameters, and work function data for HAT/Ag(111) (PDF)

## ABBREVIATIONS

BTB: 1,3,5-benzenetribenzoic acid
DFT: density functional theory
Gr: graphene
HAT: 2,3,6,7,10,11-hexmethoxytriphenylene
HATCN: 2,3,6,7,10,11-hexacyanohexaazatriphenylene
HOMO: highest occupied molecular orbital
IP: ionization potential
LEED: low-energy electron diffraction
PTCDA: perylenetetracarboxylic dianhydride
PTCDI: perylenetetracarboxylic diimide
SECO: secondary electron cutoff
STM: scanning tunneling microscopy
TCA: benzene-1,3,5-tricarboxaldehyde
TMA: trimesic acid
UHV: ultrahigh vacuum
UPS: ultraviolet photoelectron spectroscopy
XPS: X-ray photoelectron spectroscopy.
