# Supplementary material for: Comparing Adsorption of an Electron-Rich Triphenylene Derivative: Metallic vs Graphitic Surfaces
Source: J Phys Chem C Nanomater Interfaces. 2024 Jun 19;128(26):11014–23. doi: 10.1021/acs.jpcc.4c02376 (PMC11229062; doi:10.1021/acs.jpcc.4c02376)
Supplement: Supplementary file 1 — jp4c02376_si_001.pdf [file jp4c02376_si_001.pdf]

Supplementary information

Comparing Adsorption of Triphenylene Derivatives: Metallic vs. Graphitic Surfaces

Joris de la Rie[a], Qiankun Wang[a], Mihaela Enache[a], Milan Kivala[b], Meike Stöhr[a]

[a] Zernike Institute for Advanced Materials, University of Groningen, Nijenborgh 4, 9747 AG Groningen (The Netherlands)

[b] Institute of Organic Chemistry, University of Heidelberg, Im Neuenheimer Feld 270, 69120 Heidelberg (Germany)

STM

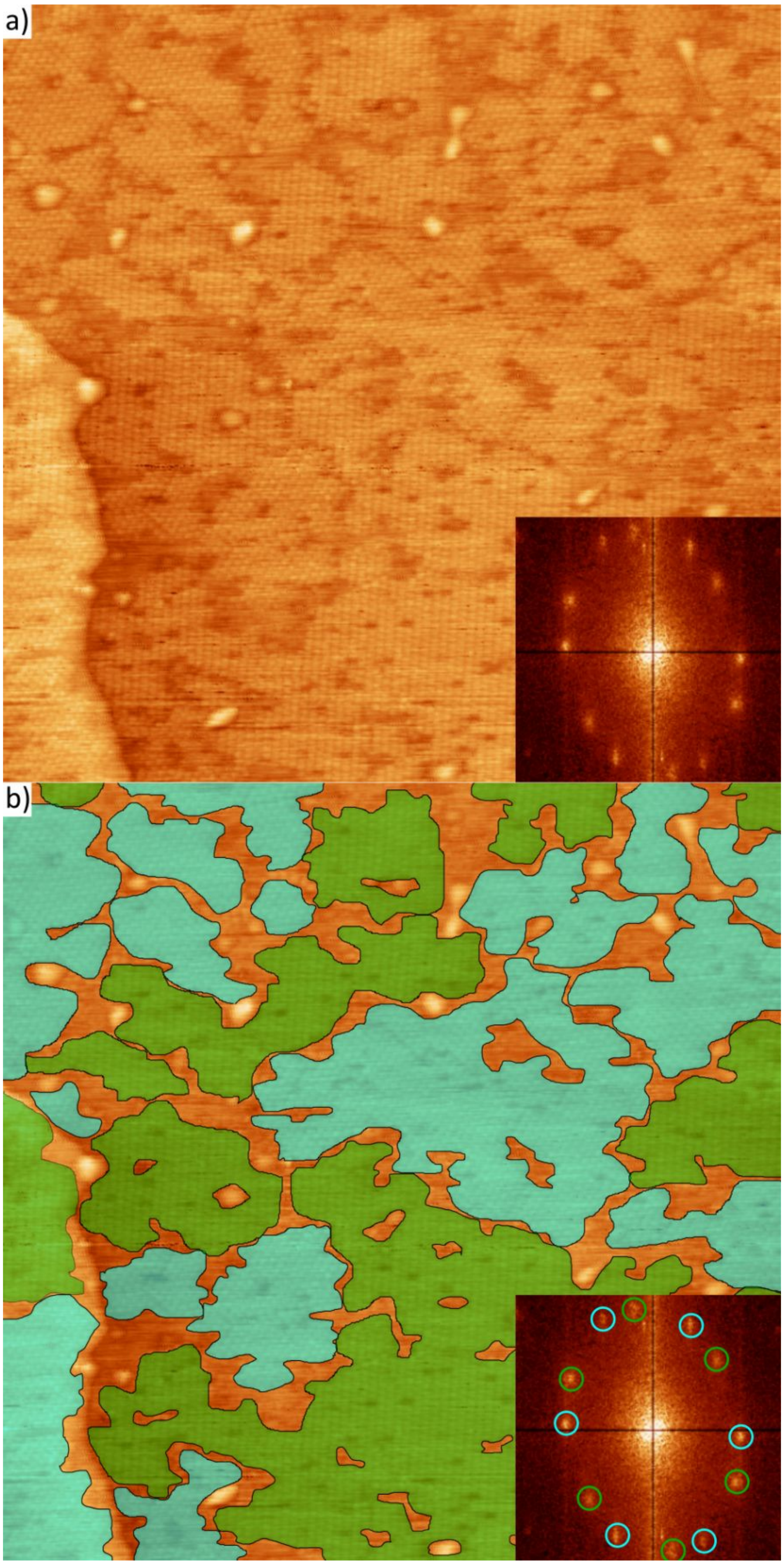

Figure S1: Overview STM image of HAT on graphene/Ni(111). a) STM image (same image as Figure 1a in the manuscript, 100x100 nm^2, 1.56 V, 20 pA). The inset shows the fast Fourier transformation (FFT). b) The same image as a), with an overlay marking the two mirror domains of HAT in green and blue. The corresponding spots in the FFT (see inset) are marked in the same colour.

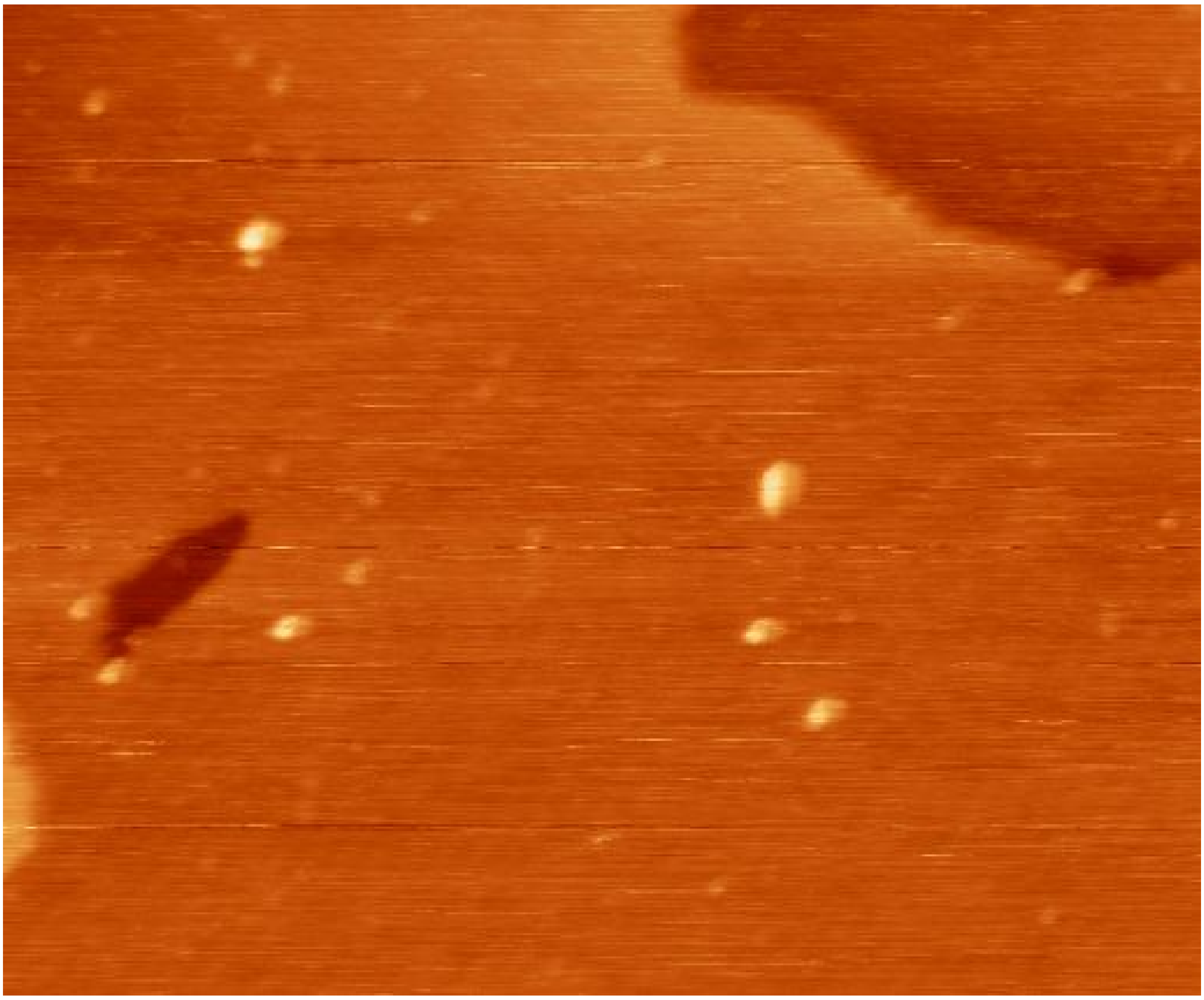

Figure S2: Overview STM image of graphene/Ni(111), showing clearly the graphene growth defects and contaminations from the Ni(111) substrate. Size 200x165.7nm^2; bias -1.50 V; current setpoint 20 pA.

LEED

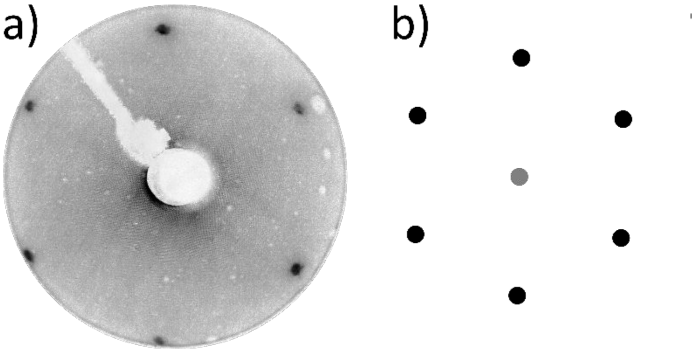

Figure S3: LEED pattern of graphene on Ni(111). a) Experimental LEED pattern obtained at 80.8 eV. The (00) spot is blocked by the electron gun. b) Simulated LEED pattern, the (00) spot in grey and the Gr/Ni(111) spots in black.

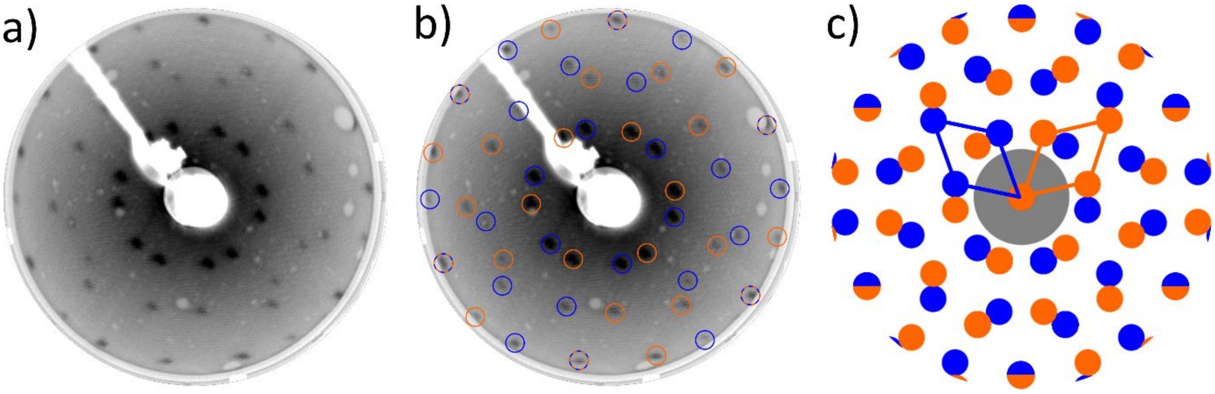

Figure S4: LEED pattern of HAT on graphene on Ni(111). a) Experimental LEED pattern obtained from 1.1 monolayer of HAT on graphene on Ni(111) at 18 eV. b) The same pattern as in a), with the spots from two HAT mirror domains marked with orange and blue circles. Spots that occur in both domains and overlap are marked with an orange-blue circle. c) Simulated pattern. The grey spot in the centre is the (00) spot, the orange and blue spots are due to the two mirror domains of HAT. Overlapping spots are marked by a half blue, half orange circle. The diamonds indicate the unit cell of the reciprocal lattice of each the domain.

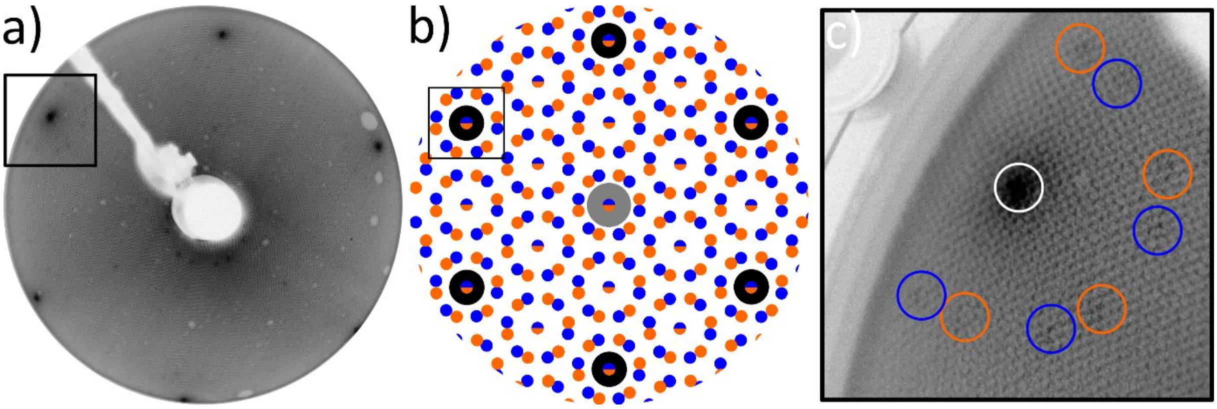

Figure S5: LEED pattern of HAT on graphene on Ni(111). Experimental LEED pattern obtained from 1.1 monolayer of HAT on graphene on Ni(111) at 75 eV. b) Simulated LEED pattern. The grey spot in the centre indicates the (00) spot, the black spots at the edges indicate the 1st order of Gr/Ni(111) spots. The orange and blue spots are due to the two mirror domains of HAT. c) A close-up of the substrate (10) spot marked in a) and b) by a black rectangle, and the ring of HAT spots surrounding it. The spots are marked by rings. The rings are colour-coded as in b) except for the substrate spot which is marked in white.

XPS

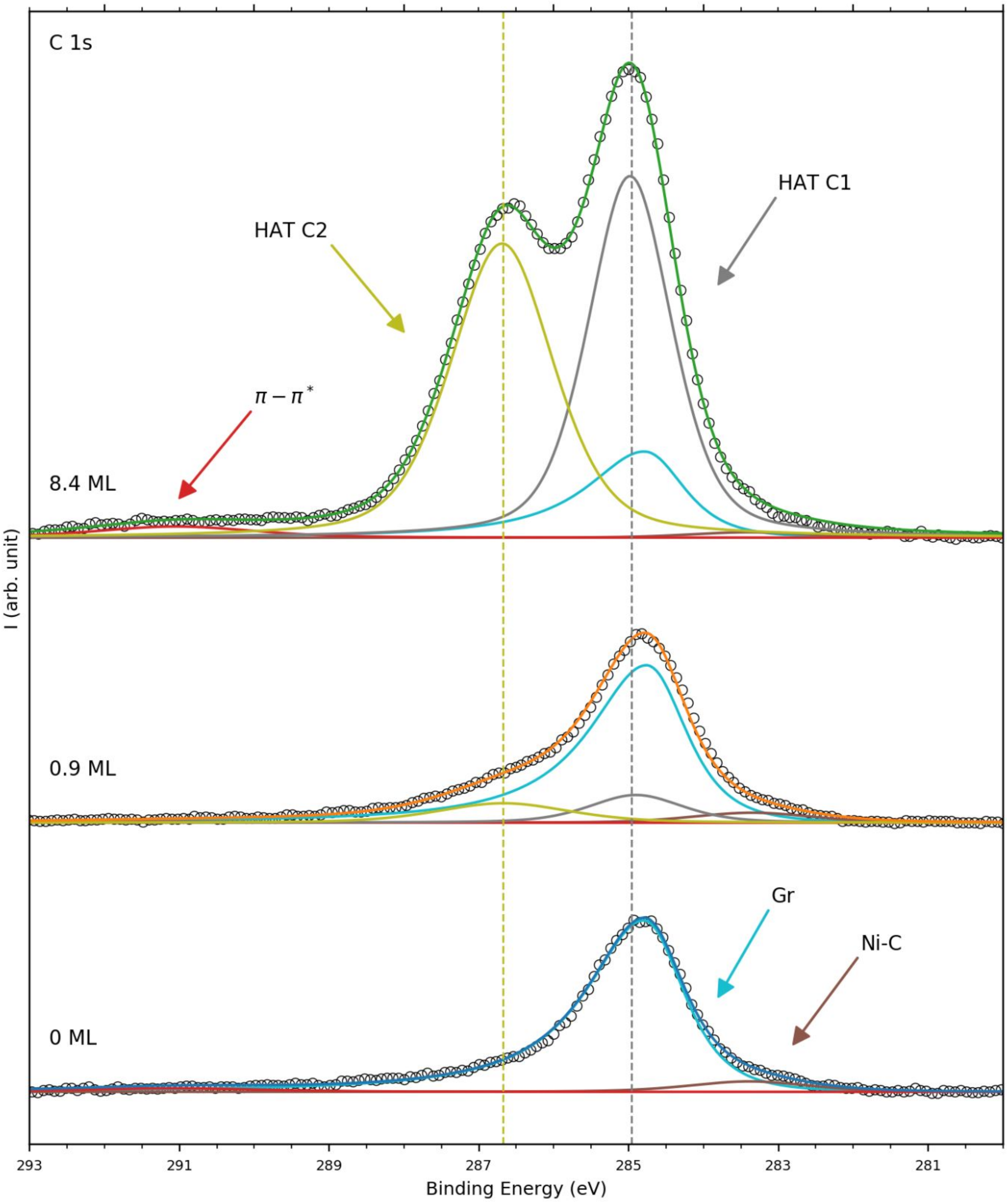

Figure S6: C 1s core level spectra for for Gr/Ni(111) (blue), monolayer HAT (orange) and multilayer HAT (green). The Gr/Ni(111) spectrum is fitted with three peaks: graphene (Gr, cyan), nickel carbide (Ni-C, brown) and a π-π* shakeup satellite (red). For the mono- and multilayer spectra two peaks for the HAT molecule are introduced, C1 (grey) and C2 (yellow), the position of which is indicated by the dashed lines.

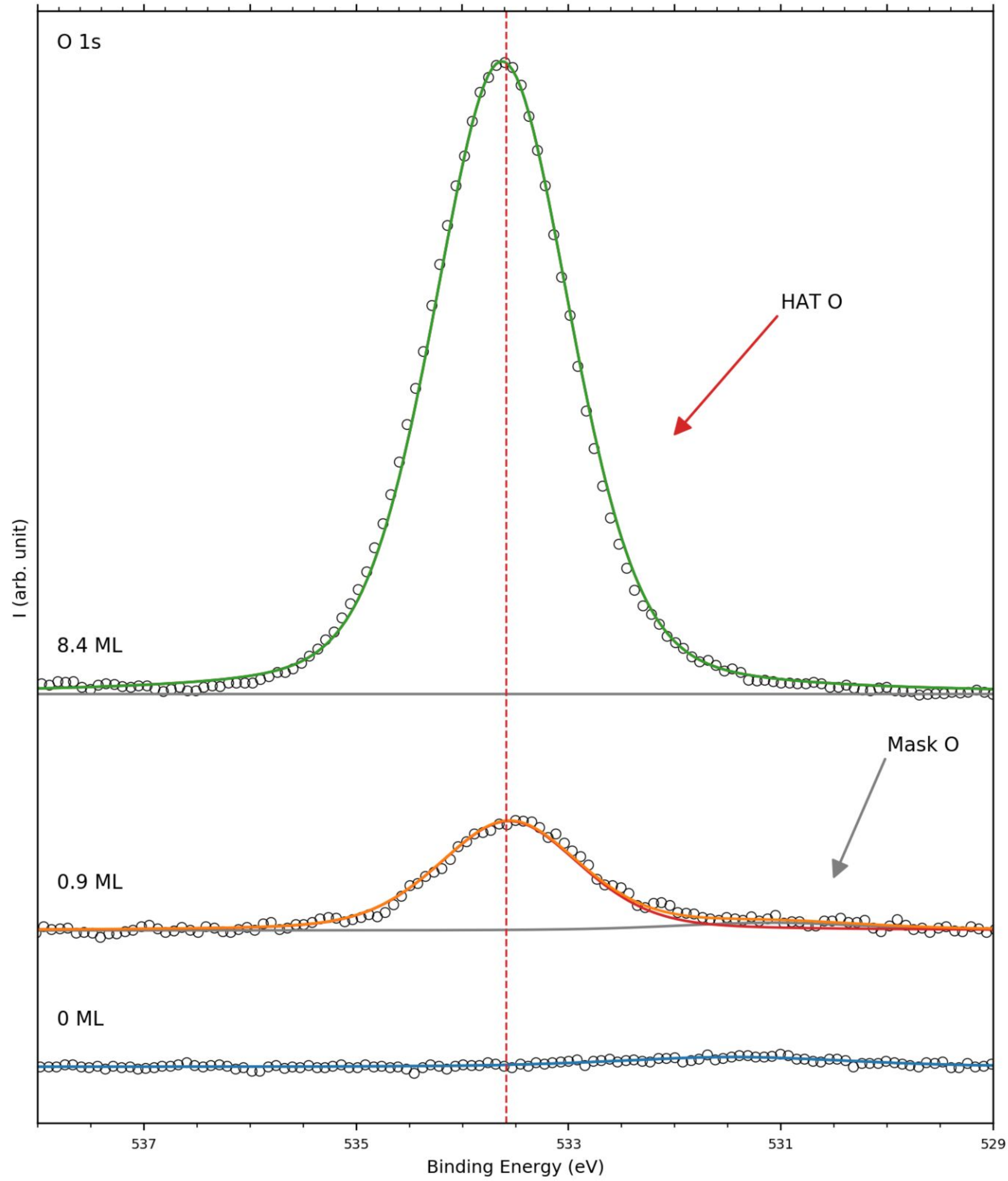

Figure S7: O 1s core level spectra for Gr/Ni(111) (blue), monolayer HAT (orange) and multilayer HAT (green). The spectra are fitted with two peaks: one for the molecular oxygen (red) and one for signal from the crystal mask (grey). The position of the molecular peak is indicated by a dashed red line.

	C 1s					O 1s	
	Graphen e	Carbide	π-π* satellite	HAT C1	HAT C2	HAT O	Crystal mask
Gr/Ni(111)	284.8 eV 1.2 eV 93 %	283.4 eV 1.9 eV 6%	291.1 eV 3.0 eV 3%	-	-	-	531.1 eV 2.6 eV 100%
Monolayer HAT	284.8 eV 1.2 eV 72%	283.4 eV 1.9 eV 5%	291.0 eV 3.2 eV 1%	284.9 eV 1.5 eV 11%	286.7 eV 2.1 eV 11%	533.6 eV 1.6 eV 91%	531.1 eV 2.14 eV 9%
Multilayer HAT	284.8 eV 1.2 eV 13%	283.4 eV 1.9 eV 1%	291.1 eV 2.5 eV 2.5%	285.0 eV 1.3 eV 42%	286.7 eV 1.6 eV 42%	533.6 eV 1.5 eV 100%	531.1 eV 2.14 eV 0%

Table S1: XPS binding energies (eV), FWHM (eV) and relative area (%) for the C 1s and O 1s core levels for graphene/Ni(111), 0.9 ML HAT and 8.4 ML HAT for the data shown in Figure 2.

UPS

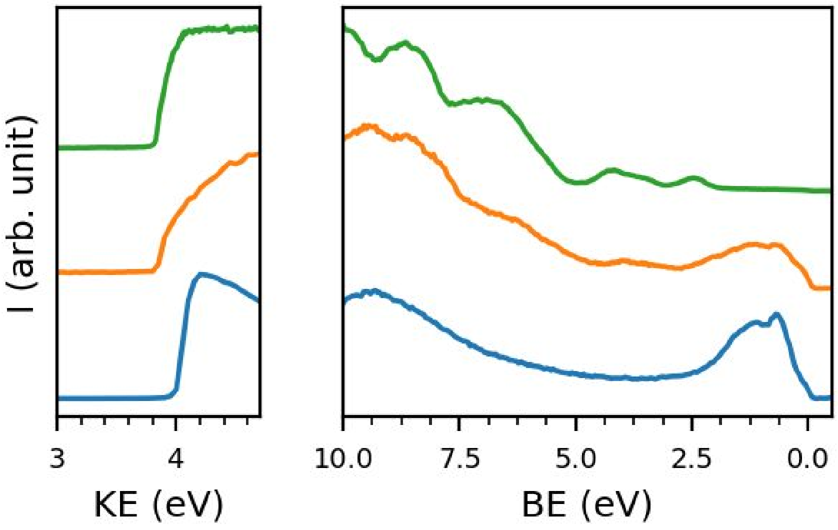

Figure S8: UPS data for HAT on Gr/Ni(111), measured with He I (21.2 eV) light. Pristine graphene on Ni(111) (blue), 0.9 ML HAT (orange) and 8.4 ML HAT (green). a) Secondary electron cut-off, b) valence band spectra.

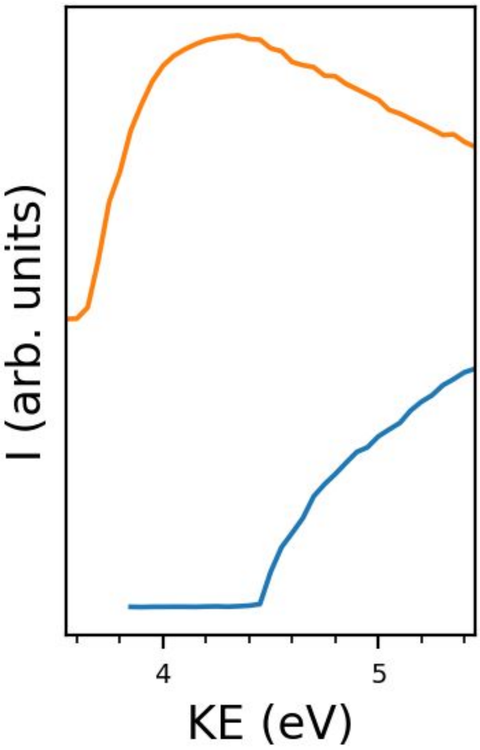

Figure S9: Secondary electron cut-off for HAT on Ag(111), measured with He I (21.2 eV) light at a sample bias of -5 V. Clean Ag(111) (blue, work function 4.5 eV) and one monolayer of HAT on Ag(111) (orange, work function 3.7).

Calculated adsorption energies for molecules discussed in the manuscript

Molecule	Substrate	Adsorption energy (per molecule, eV)	Molecule-molecule interaction energy (per molecule, eV)	Molecule-substrate interaction energy (per molecule, eV)
HAT[1]	Ag(111)	2.27		
TMA (chickenwire)[2]	Graphene	2.19	0.89	1.3
TMA (flower)[2]	Graphene	2.03	0.85	1.18
PTCDA (single molecule)[3]	Graphene	1.2	0	
PTCDA (network)[3]	Graphene	0.83	0.78	
C_{60}[4]	Graphene/Cu(111)	1.787		
C_{60}[4]	Gas phase		0.796	
TCNQ[5]	Graphene	1.43	0.51	
F_4-TCNQ[5]	Graphene	1.26	0.1	

Table S2: Adsorption energy; intermolecular interaction energy and molecule-substrate interaction energy for molecules on graphene discussed in the manuscript and for HAT on Ag(111). Calculations are for a single layer of molecules on graphene (no support simulated), unless otherwise noted. Adsorption energy is the total energy gained by adsorbing a full layer of molecules on the substrate, per molecule; molecule-molecule interaction energy is the amount of this that is due to molecule-molecule interactions.

References

(1) Müller, K.; Schmidt, N.; Link, S.; Riedel, R.; Bock, J.; Malone, W.; Lasri, K.; Kara, A.; Starke, U.; Kivala, M.; et al. Triphenylene-Derived Electron Acceptors and Donors on Ag(111): Formation of Intermolecular Charge-Transfer Complexes with Common Unoccupied Molecular States. *Small* **2019**, *15* (33), 1901741. https://doi.org/10.1002/smll.201901741.

(2) Rochefort, A.; Shayeganfar, F. Electronic Properties of Self-Assembled Trimesic Acid Monolayer on Graphene. *Langmuir,* **2014,** *30* (32), 9707-9716. https://doi.org/10.1021/la501619b

(3) Tian, X. Q.; Xu, J. B.; Wang, X. M. Self-Assembly of PTCDA Ultrathin Films on Graphene: Structural Phase Transition and Charge Transfer Saturation. *J. Phys. Chem. C* **2010**, *114* (49), 20917–20924. https://doi.org/10.1021/jp1031674.

(4) Minbok, J.; Shin, D.; Sohn, S.-D.; Kwon; S.-Y.; Park, N.; Shin, H.-J. Atomically resolved orientational ordering of C60 molecules on epitaxial graphene on Cu(111). *Nanoscale*, **2014**, *6* (20), 11835-11840 https://doi.org/10.1039/c4nr03249g

(5) Yang, S.; Jiang, Y.; Li, S.; Liu, W. Many-body dispersion effects on the binding of TCNQ and F4-TCNQ with graphene. *Carbon*, **2017**, *111* (2017), 513-518. http://dx.doi.org/10.1016/j.carbon.2016.10.024
